# Levocabastine eye drops are effective and well tolerated for the treatment of allergic conjunctivitis in children

**DOI:** 10.1155/S0962935195000792

**Published:** 1995-05

**Authors:** Brunello Wüthrich, Martin Gerber

**Affiliations:** 1 Allergy Unit Department of Dermatology University Hospital Zürich Switzerland; 2 janssen Research Foundation Sihlbruggstrasse 111 Postfach 58 Baar CH-6341 Switzerland

## Abstract

This open-label, prospective, multicentre, 4-week trial was undertaken to assess the efficacy and tolerability of twice daily levocabastine eye drops (0.5 mg/ml), with sodium cromoglycate nasal spray for the relief of concurrent nasal symptoms if required, in a total of 233 children with seasonal allergic conjunctivitis. No correlation between efficacy, tolerability and age was found. Investigator assessments revealed that the total severity of ocular symptoms decreased by 84 ± 34% in patients < 12 years and 85 ± 30% in those ≥ 12 years, with corresponding reductions in the total severity of ocular findings of 84% in both patient groups over the 4-week treatment period. Global assessments of therapeutic efficacy revealed the effect of therapy on ocular symptoms to be excellent or good in 81% of patients < 12 years and 82% of those ≥ 12 years after 2 weeks of treatment, with corresponding values at the end of the trial of 88% and 82% in the two groups, respectively. Treatment tolerability was considered to be excellent or good by 94% of patients overall. Application site reactions were the most common adverse event associated with ocular levocabastine, occurring in 13% of patients < 12 years and 9% of those ≥ 12 years. Twice daily levocabastine eye drops therefore appear to be effective and well tolerated for the treatment of seasonal allergic conjunctivitis in children.


Mediators of Inflammation 4, S16–S20 (1995)

### Levocabastine eye drops are effective and well tolerated for the treatment of allergic conjunctivitis in children

Brunello Wüthrich[1] and Martin Gerber[2,CA]

[1]Allergy Unit, Department of Dermatology, University Hospital, Zürich, Switzerland;
[2]Janssen Research Foundation, Sihlbruggstrasse 111, Postfach 58, CH-6341 Baar, Switzerland

[CA]Corresponding Author

**THIS open-label, prospective, multicentre, 4-week trial was undertaken to assess the efficacy and tolerability of twice daily levocabastine eye drops (0.5 mg/ml), with sodium cromoglycate nasal spray for the relief of concurrent nasal symptoms if required, in a total of 233 children with seasonal allergic conjunctivitis. No correlation between efficacy, tolerability and age was found. Investigator assessments revealed that the total severity of ocular symptoms decreased by 84 ± 34% in patients < 12 years and 85 ± 30% in those ⩾ 12 years, with corresponding reductions in the total severity of ocular findings of 84% in both patient groups over the 4-week treatment period. Global assessments of therapeutic efficacy revealed the effect of therapy on ocular symptoms to be excellent or good in 81% of patients < 12 years and 82% of those ⩾ 12 years after 2 weeks of treatment, with corresponding values at the end of the trial of 88% and 82% in the two groups, respectively. Treatment tolerability was considered to be excellent or good by 94% of patients overall. Application site reactions were the most common adverse event associated with ocular levocabastine, occurring in 13% of patients < 12 years and 9% of those ⩾ 12 years. Twice daily levocabastine eye drops therefore appear to be effective and well tolerated for the treatment of seasonal allergic conjunctivitis in children.**

**Key words:** Allergic conjunctivitis, Children, $H_1$-receptor antagonist, Levocabastine, Topical antihistamine

### Introduction

Levocabastine is a novel selective $H_1$-receptor antagonist which has been specifically developed as eye drops and nasal spray for the topical treatment of allergic rhinoconjunctivitis.[1] Levocabastine is the most potent antihistamine available to date, being some 15 000 times more potent than chlorpheniramine on a molar basis and expressing antihistaminic activity at doses as low as 0.002 mg/kg.[2] Onset of action is rapid, typically occurring within minutes of instillation, with duration of effect sufficiently long to permit a convenient twice daily dosing regimen.[3,4]

The efficacy and tolerability of levocabastine eye drops in the treatment of allergic conjunctivitis in adults is well documented.[5] Comparative studies have shown that levocabastine eye drops administered twice daily are at least as effective as standard daily doses of oral antihistamines[6–10] and significantly more effective than sodium cromoglycate four times daily.[11,12] Levocabastine eye drops have also been shown to be significantly more effective than the topical antihistamine/vasoconstrictor combination, antazoline/naphazoline, for the treatment of ocular symptoms,[13] with a tolerability profile comparable with that of sodium cromoglycate or placebo.[14]

Preliminary studies in children, involving a total of 157 patients, have shown that levocabastine eye drops administered twice daily are at least as effective and well-tolerated as sodium cromoglycate four times daily for the treatment of allergic conjunctivitis, both as single agent therapy[15,16] and as an adjunct to oral antihistamine therapy.[17] The present study was undertaken to assess the efficacy and tolerability of ocular levocabastine in the routine treatment of seasonal allergic conjunctivitis in a much larger group of children and adolescents. Assessment of any correlation between efficacy, tolerability and age (< 12 years and ⩾ 12 years) was a secondary aim.

### Materials and Methods

*Study design:* Children and adolescents (aged 5 to 16 years) with a history of seasonal allergic conjunctivitis were eligible for inclusion into this open-label, prospective, multicentre trial which

© 1995 Rapid Communications of Oxford Ltd was conducted during the hay fever seasons of 1992 and 1993. All were required to have a minimum of two characteristic symptoms of allergic conjunctivitis of at least moderate severity at the time of entry into the trial. Patients with soft contact lenses and concurrent disorders which might have interfered with evaluation of the study medication were excluded from participation. In addition, patients were required to discontinue use of other antiallergic medication (for example, oral antihistamines, vasoconstrictors or corticosteroids) prior to study entry.

All patients received levocabastine eye drops (0.5 mg/ml), one drop in each eye, twice daily for a total of 4 weeks. Sodium cromoglycate nasal spray (20 mg/ml; one spray four times daily) was provided for use only in patients in whom concurrent nasal symptoms became moderate or severe. Antazoline and tetryzoline eye drops or sodium cromoglycate plus xylometazoline could be used in patients with severe symptoms with a maximum treatment duration of 3 days. No other rescue medication was provided and use of other medications which could interfere with the evaluation of the study drug was not permitted during the trial period.

The study design was approved by the local ethics committee and all children and their parents provided informed consent.

*Efficacy assessments:* Ocular symptoms (pruritus, lacrimation, photophobia and pain), ocular signs (conjunctival erythema, conjunctival oedema and eyelid oedema) and nasal symptoms (congestion, rhinorrhoea, pruritis and sneezing) were assessed by the investigator at the start of the trial to obtain baseline measurements and then after 2 and 4 weeks of treatment, as well as by the patients (helped by their parents if necessary) on a daily basis, using a 4-point scale (0 = absent, 1 = mild, 2 = moderate, 3 = severe). In addition, the investigator provided a global evaluation of treatment efficacy for both ocular and nasal symptoms, as well as treatment tolerability, after 2 weeks of treatment and at the end of the trial, rating therapy as excellent, good, satisfactory or unsatisfactory.

*Statistical analysis:* Patients were divided into two subgroups according to age: $< 12$ years (children) and $\geqslant 12$ years (adolescents). In addition to the mean severity for each of the individual symptoms listed above, the following parameters were calculated and analysed: the mean total severity score for ocular symptoms, the mean total severity score for ocular findings, and the mean total severity score for nasal symptoms. Intergroup comparisons were made using Student's $t$-test for parametric data or the chi-squared test for non-parametric data (5% level of significance).

### Results

A total of 233 patients were enrolled in this study (177 children and 56 adolescents) by 34 paediatricians. Although all patients are included in the tolerability analysis, 27 have been excluded from the efficacy analysis (21 due to insufficient symptom severity at baseline, one due to age ($<$ 5 years), two due to a combination of these two factors and three due to non-compliance with the study protocol/early drop-out). Patient demographics for the remaining 206 patients who were included in the efficacy analysis are shown in Table 1. As expected, the mean age, weight and height differed significantly between the two patient groups ($p < 0.001$). In addition, patients $<$ 12 years of age mostly lived in rural environments, whilst those $\geqslant$ 12 years were predominantly from urban areas (chi-squared, $p < 0.01$). Symptom severity at baseline was generally

**Table 1.** Patient demography

| | < 12 years | ≥ 12 years |
|---|---|---|
| Number of patients (M/F) | 157 (103/54) | 49 (32/17) |
| Mean age in years (range) | 7.6 (4–11)*** | 13.4 (12–16) |
| Mean weight in kg (range) | 27.7 (13–56)*** | 51.1 (32–115) |
| Mean height in cm (range) | 127.9 (100–167)*** | 161.1 (143–187) |

***$p < 0.001$.

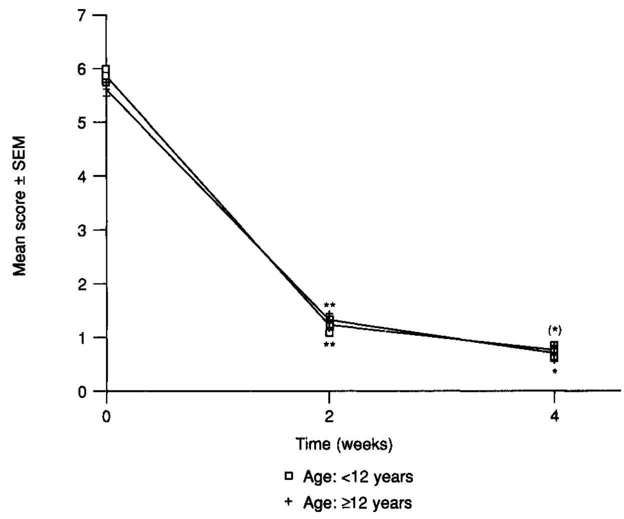

FIG. 1. Total symptom severity scores for ocular symptoms during the 4-week treatment period. **$p < 0.01$ compared with baseline values; *$p < 0.05$ and (*)$0.05 < p < 0.1$ week 4 compared with week 2.

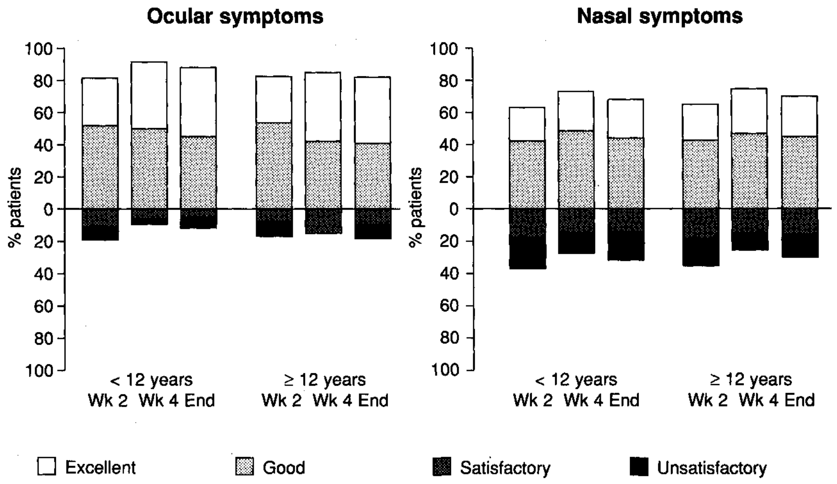

FIG. 2. Investigator assessments of global therapeutic efficacy for ocular and nasal symptoms after 2 and 4 weeks of treatment and at study end-point.

comparable, although ocular findings were significantly more severe in patients < 12 years compared with those ⩾ 12 years ($p < 0.05$), while the severity of rhinorrhoea was greater in the older patient group ($p < 0.01$). In all, 140 patients were eligible for treatment with sodium cromoglycate nasal spray.

Significant reductions in symptom severity compared with baseline values were apparent in both patient groups within 2 weeks of initiation of therapy for all parameters evaluated ($p < 0.01$). As shown in Fig. 1, the mean total severity score for ocular symptoms decreased by 84 ± 34% in patients < 12 years and 85 ± 30% in those ⩾ 12 years over the 4-week treatment period, with a 84 ± 40% reduction in the total severity of ocular signs in both patient groups over this period of time.

Pruritus was the most frequent ocular symptom at baseline reported as moderate to severe by 89% of patients < 12 years and 92% of those ⩾ 12 years, but moderate to severe ocular pruritus was only present in 7.7% and 6.3% of patients in the two groups, respectively, at the end of the trial (end-point values). Similarly, the incidence of patients with moderate to severe conjunctival erythema, the most severe ocular sign at baseline, was reduced from 89% to 9% in patients < 12 years and from 88% to 8% in those ⩾ 12 years over the 4-week treatment period (end-point values) ($p < 0.001$).

Analysis of the data generated in the patients' diaries revealed similar findings. At the end of the trial, the mean reduction in total symptom severity from baseline was 73 ± 36% in patients < 12 years and 65 ± 40% in those ⩾ 12 years (baseline scores 2.2 ± 0.7 in patients < 12 years and 2.0 ± 0.7 in patients ⩾ 12 years; 0 = absent, 3 = severe).

Investigator assessments of global therapeutic efficacy are shown in Fig. 2. After 2 weeks of treatment, the effect of therapy on ocular symptoms was considered to be excellent or good in 81% of patients < 12 years and 82% of those ⩾ 12 years. The corresponding values at end-point were 88% and 82% in the two groups, respectively.

Global therapeutic efficacy for nasal symptoms was considered to be excellent or good in 63% of patients < 12 years after 2 weeks of treatment and 68% at study end-point. Corresponding values for patients ⩾ 12 years were 65% and 71%, respectively, at these times.

Global evaluations of treatment tolerability in the patients included in the efficacy analysis are shown in Fig. 3. At the end of treatment, 94% of patients in both age groups considered tolerability to be excellent or good. Adverse events

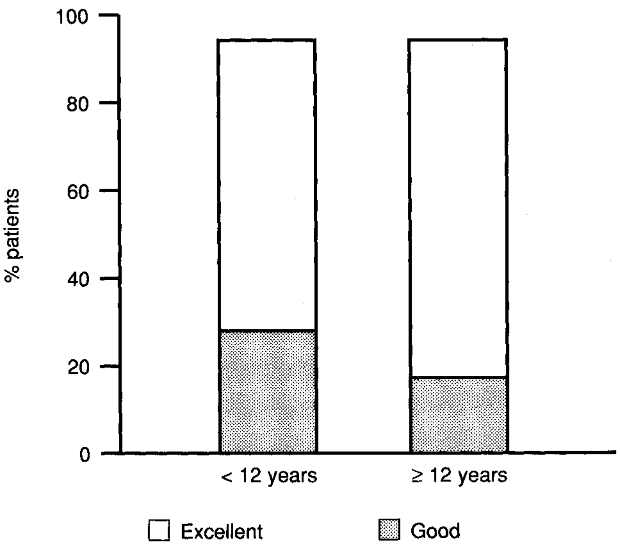

FIG. 3. Investigator assessments of global treatment tolerability at study end-point.

**Table 2.** Incidence of most common adverse events (reported by at least two patients per group)

| | < 12 years | ⩾ 12 years |
|---|---|---|
| Total number of patients | 177 | 56 |
| Total number of patients reporting adverse events | 33 | 7 |
| Ocular adverse events | | |
| Burning | 17 | 5 |
| Irritation | 5 | 0 |
| Pruritis | 5 | 0 |
| Erythema | 2 | 0 |
| Oedema | 2 | 0 |
| Others | 3 | 0 |

were reported by 33 (18.6%) of all patients < 12 years and 7 (12.5%) of those ⩾ 12 years, with no statistically significant intergroup differences in terms of severity or type (Table 2). In all, four patients (three who were < 12 years and one of ⩾ 12 years) discontinued or interrupted treatment due to adverse events. Reasons for treatment withdrawal in patients < 12 years were ocular burning in two patients and erythema rash in another. Treatment was interrupted in one patient ⩾ 12 years due to diarrhoea. The most common adverse events were ocular burning (occurring in 9.6% of patients < 12 years and 8.9% of those ⩾ 12 years) and/or ocular irritation (reported by 2.8% of patients < 12 years).

### Discussion

The results of this open-label, prospective, multicentre trial clearly demonstrate that levocabastine eye drops are effective and well-tolerated for the treatment of allergic conjunctivitis in children, with no apparent correlation between efficacy, tolerability and age. The severity of all symptoms was significantly reduced from baseline values after 2 weeks of treatment for all parameters evaluated, with further reductions apparent by the end of the trial. Overall, response rates were found to be generally comparable with those reported in adults.[5–13] These findings are supported by those of another recent paediatric study undertaken to compare the efficacy and tolerability of topical levocabastine with that of sodium cromoglycate.[18]

Drug tolerability is a key factor determining choice of therapy in children. In this study, levocabastine was found to be well-tolerated with adverse events reported in 18.6% of children and 12.5% of adolescents. As might be expected from the route of drug administration, application site reactions (ocular burning and irritation) were the most common adverse events reported during treatment with levocabastine eye drops, occurring in 12.4% and 8.9% of patients in the two groups, respectively. Studies in adults have shown that the adverse effect profile of topical levocabastine is comparable with that of sodium cromoglycate and placebo with ocular irritation reported in 14% of patients treated with levocabastine eye drops, to date, compared with 15% for placebo-treated controls.[19]

In summary, twice daily treatment with levocabastine eye drops appears to be effective and well tolerated for the treatment of seasonal allergic conjunctivitis in children. Furthermore, comparison of the available data suggests that results of studies undertaken to assess the efficacy and tolerability of topical levocabastine in adults can be generalized to paediatric patients. Clinical experience to date therefore suggests that levocabastine eye drops are an attractive primary option for the treatment of seasonal allergic conjunctivitis in this patient population.

### References

1. Dechant KL, Goa KL. Levocabastine: a review of its pharmacological properties and therapeutic potential in allergic rhinitis and conjunctivitis. *Drugs* 1991; **41:** 202 –224.
2. Van Wauwe JP. Animal pharmacology of levocabastine: a new type of $H_1$-antihistamine well-suited for topical application. In: Mygind N, Naclerio RM, eds. *Rhinoconjunctivitis: New Perspectives in Topical Treatment of Seasonal Allergic Rhinitis.* Proceedings of the XIIIth International Congress of Allergology and Clinical Immunology. Gottingen: Hogrefe and Huber, 1989; 27–34.
3. Stokes TC, Feinberg G. Rapid onset of action of levocabastine eye drops in histamine-induced conjunctivitis. *Clin Exp Allergy* 1993; **23:** 791–794.
4. Tomiyama S, Ohnish M, Okuda M. The dose and duration of effect of levocabastine, a new topical $H_1$-receptor antagonist on nasal provocation reaction to allergen. *Am J Rhinol* 1993; **7:** 85–88.
5. Abelson MB, Weintraub D. Levocabastine eye drops: a new approach for the treatment of acute allergic conjunctivitis. *Eur J Ophthalmol* 1994; **4:** 91–101.
6. The Livostin Study Group. A comparison of topical levocabastine and oral terfenadine in the treatment of allergic rhinoconjunctivitis. *Allergy* 1993; **48:** 530–534.
7. Søhoel P, Freng BA, Kramer J, *et al.* Topical levocabastine compared with oral terfenadine for the treatment of seasonal allergic rhinoconjunctivitis. *J Allergy Clin Immunol* 1993; **92:** 73–81.
8. Bahmer FA, Ruprecht KW. Safety and efficacy of topical levocabastine compared with oral terfenadine. *Ann Allergy* 1994; **72:** 429–434.
9. The Swedish GP Allergy Team. Topical levocabastine compared with oral loratadine for the treatment of seasonal allergic rhinoconjunctivitis. *Allergy* 1994; **49:** 611–615.
10. Drouin MA, Yang WH, Horak F. Faster onset of action with topical levocabastine than with oral cetirizine. *Med Inflamm* 1995; **4 (1):** S5–S10.
11. Azevedo M, Castel-Branco MG, Ferrez Oliveira J, *et al.* Double-blind comparison of levocabastine eye drops with sodium cromoglycate and placebo in the treatment of seasonal allergic conjunctivitis. *Clin Exp Allergy* 1991; **21:** 689–694.
12. Davies BH, Mullins J. Topical levocabastine is more effective than sodium cromoglycate for the prophylaxis and treatment of seasonal allergic conjunctivitis. *Allergy* 1993; **48:** 519–524.
13. Bende M, Pipkorn U. Topical levocabastine, a selective $H_1$-antagonist, in seasonal allergic rhinoconjunctivitis. *Allergy* 1987; **42:** 512–515.
14. Janssens M, Blockhuys S. Tolerability of levocabastine eye drops. *Doc Ophthalmol* 1993; **84:** 111–118.
15. Odelram H, Bjorkstén B, Af Klercker T, *et al.* Topical levocabastine versus sodium cromoglycate in allergic conjunctivitis. *Allergy* 1989; **44:** 432–436.
16. Möller C, Blychert L-O. Levocabastine eye drops in comparison with cromoglycate in the treatment of conjunctivitis in children with birch pollinosis. *Pediatr Allergy Immunol* 1990; **1:** 87–89.
17. Njaa F, Baekken T, Bjaamer D, *et al.* Levocabastine compared with sodium cromoglycate eye drops in children with birch and grass pollen allergy. *Pediatr Allergy Immunol* 1992; **3:** 39–42.
18. Vermeulen J, Mercer M. Topical levocabastine is more effective than sodium cromoglycate in children with seasonal allergic rhinoconjunctivitis. *Pediatr Allergy Immunol* 1994; **5:** 209–213.
19. Howarth P. A review of the tolerability and safety of levocabastine eye drops and nasal spray. Implications for patient management. *Med Inflamm* 1995; . **4 (1):** S26–S30.

ACKNOWLEDGEMENTS. The authors wish to thank the following investigators to the study: B. Baumgartner-Lips, T. A. Berther, M. Bovet Vetter, R. Christen, V. D'Apuzzo, R. Ebner, B. Evequoz, H. Fuchs, E. Galler-Benninger, E. Gerber-Hobl, St. Gilardi, H. Häfliger, I. Högger-Imhof, J. Hofer, A. Hohl, M. Huguenin, I. Hunkeler, W. Jörg-Eidenbenz, A. Keel, H. P. Keller, H. P. Kind, P. Kindler, [illegible]. Knöpfli, R. Lejeune, M. J. Mettler, A. M. Oppikofer, L. Rützler, D. Schibler, H. Schön, S. Sichitiu, M. Terrapon, A. Weil, W. Weiss.
